# TNM8: The updated TNM classification for retinoblastoma

**Published:** 2018-06-03

**The TNM classification for retinoblastoma, which is developed by the American Joint Committee on Cancer (AJCC), has recently been updated from TNM7 to TNM8. The newest dataset for retinoblastoma from the UK's Royal College of Pathologists (RCPath), released in early 2018, reflects the changes in the TNM classification.**

**Table:** 

**Definition of primary tumour (cT)**		
cTX		Unknown evidence of intraocular tumour
cT0		No evidence of intraocular tumour
cT1		Intraocular tumour(s) with sub-retinal fluid ≤ 5mm from the base of any tumour
	cT1a	Tumours ≤ 3mm and further than 1.5 mm from the disc and fovea
	cT1b	Tumours > 3 mm or closer than 1.5 mm to the disc and fovea
cT2		Intraocular tumour(s) with retinal detachment, vitreous seeding or sub-retinal seeding
	cT2a	Sub-retinal fluid > 5 mm from the base of any tumour
	cT2b	Tumours with vitreous seeding and/or sub-retinal seeding
cT3		Advanced intraocular tumour(s)
	cT3a	Phthisis or pre-phthisis bulbi
	cT3b	Tumour invasion of the pars plana, ciliary body, lens, zonules, iris or anterior chamber
	cT3c	Raised intraocular pressure with neovascularization and/or buphthalmos
	cT3d	Hyphema and/or massive vitreous hemorrhage
	cT3e	Aseptic orbital cellulitis
cT4		Extraocular tumour(s) involving the orbit, including the optic nerve
	cT4a	Radiological evidence of retrobulbar optic nerve involvement or thickening of the optic nerve or involvement of the orbital tissues
	cT4b	Extraocular tumour clinically evident with proptosis and orbital mass
**Definition of regional lymph nodes (cN)**		
cNX		Regional lymph nodes cannot be assessed
cN0		No regional lymph nodes involvement
cN1		Evidence of preauricular, submandibular, and cervical lymph node involvement
**Definition of distant metastasis (M)**		
cM0		No signs or symptoms of intracranial or distant metastasis
cM1		Distant metastasis without microscopic confirmation
	cM1a	Tumour(s) involving any distant site (e.g. bone marrow, liver) on clinical or radiological tests
	cM1b	Tumour involving the central nervous system on radiological imaging (not including trilateral retinoblastoma)
pM1		Distant metastasis with microscopic confirmation
	pM1a	Histopathological confirmation of tumour at any distant site (e.g. bone marrow, liver, or other)
	pM1b	Histopathological confirmation of tumour in the cerebrospinal fluid or CNS parenchyma
**Definition of heritable trait (H)**		
HX		Unknown or insufficient evidence of a constitutional RB1 gene mutation
H0		Normal RB1 alleles in blood tested with demonstrated high sensitivity assays
H1		Bilateral retinoblastoma, retinoblastoma with an intracranial CNS midline embryonic tumour (i.e. trilateral retinoblastoma), patient with family history of retinoblastoma, or molecular definition of constitutional RB1 gene mutation

